# Resistance against *Orobanche crenata* in Bitter Vetch (*Vicia ervilia*) Germplasm Based on Reduced Induction of *Orobanche* Germination

**DOI:** 10.3390/plants10020348

**Published:** 2021-02-12

**Authors:** Clara Isabel González-Verdejo, Mónica Fernández-Aparicio, Eva María Córdoba, Juan Antonio López-Ráez, Salvador Nadal

**Affiliations:** 1 IFAPA Centro Alameda del Obispo, Área de Genómica y Biotecnología, Apdo. 3092, 14080 Córdoba, Spain; evam.cordoba@juntadeandalucia.es (E.M.C.); salvador.nadal@juntadeandalucia.es (S.N.); 2 Instituto de Agricultura Sostenible-CSIC, Avda. Menéndez Pidal s/n, 14004 Córdoba, Spain; monica.fernandez@ias.csic.es; 3 Estación Experimental del Zaidín-CSIC, Profesor Albareda 1, 18100 Granada, Spain; juan.lopezraez@eez.csic.es

**Keywords:** phytogenetic resources, parasitic weeds, broomrape, legumes, breeding, low germination induction, sustainable crop protection

## Abstract

Bitter vetch (*Vicia ervilia* (L.) Willd.) is a legume well adapted to cultivation in marginal areas, being an important source of protein for animal feed in low input cropping systems. Surprisingly, it is an underutilized crop as it could be a good alternative to increase the sustainability of extensive rainfed cropping systems. In Mediterranean rainfed cropping systems, the productivity of bitter vetch is severely reduced by the parasitic weed species *Orobanche crenata* (Forsk). To date, few resistant bitter vetch genotypes have been identified. *O. crenata* infection process initiates with the recognition of germination factors exuded by roots of susceptible hosts. In this work, the interaction of a collection of bitter vetch accessions and *O. crenata* has been analyzed in order to discover accessions with low germination induction activity. Through a combination of field and rhizotron experiments, two bitter vetch accessions were selected showing low germination-induction activity, which resulted in less infection. In addition, in vitro germination assays revealed that the low germination activity was due to low exudation of germination factors and not due to the exudation of germination inhibitors. The selected low germination-inducers genotypes could be the basis for a new breeding program generating locally adapted alternatives with resistance to *O. crenata*.

## 1. Introduction

Bitter vetch (*Vicia ervilia* (L.) Willd.) is one of the oldest cultivated grain legume crops [1], whose origin is located in the Mediterranean and Middle East area [2]. It is grown as a forage and grain crop [3]. In Spain, the area of bitter vetch cultivation during 2018 reached 54,900 ha with an average of grain yield of 1150 kg/ha [4]. It is an annual, predominantly self-pollinated species, tolerant to marginal soils, and drought and cold climate conditions [5]. Despite its many advantages for cultivation in low-input cropping systems, its cultivation is in decline mainly due to the lack of investment in breeding programs to register elite cultivars [6].

The main constrain for bitter vetch cultivation in Mediterranean rainfed cropping systems is the crenata broomrape (*Orobanche crenata* Forsk.) [7]. *O. crenata* is a parasitic broomrape weed species that infects roots of different crops, mainly legume species as well as other crops including carrot or celery [8,9], causing severe yield damage [10]. Thus, there is a need to find sources of resistance in germplasm collections of crop species and their wild relatives in order to understand the responsible resistance mechanisms to facilitate the development of resistant cultivars [11]. *O. crenata* weeds are obligate parasites without root systems nor photosynthetic activity, and therefore, they are constrained to infect the root of their hosts quickly after their seeds germinate. To increase the likelihood of establishment on the host, they maintain their germination inhibited until the detection of specific germination stimulants exuded by roots of their hosts [12]. Several classes of germination stimulants are known to induce broomrape germination, with strigolactones being the main ones [13]. Additional germination factors have been reported, such as dehydrocostuslactone [14], dihydrosorgoleone [15], peagol, peagoldione, peapolyphenols A-–C, soyasapogenol B, trans-22-dehydrocampesterol [16,17,18], or isothyocyanates [19]. Because the lack of germination-induction activity in the roots of a host prevents broomrape infection, the process of host-induced parasitic germination is one of the most obvious targets for the development of resistant cultivars [20,21]. Low germination-inducers genotypes have been described previously in faba bean [22,23,24], tomato [25,26], pea [27], rice [28], sorghum [29], and sunflower [30,31]. Despite the severe damage inflicted by *O. crenata* in bitter vetch plants, few resistant genotypes have been identified so far [7]. The aim of this study was to identify differences in induction activity of *O. crenata* germination in a collection of 39 bitter vetch accessions using a combination of field, rhizotron, and in vitro experiments. The selected low germination-inducers genotypes could be the basis for a new breeding program generating locally adapted alternatives with resistance to *O. crenata* infection.

## 2. Results

### 2.1. Rhizotron Screening

A rhizotron experiment was used to study the interaction of roots of 39 bitter vetch accessions with *O. crenata* seeds under the stereomicroscope. *O. crenata* seeds interacting in close proximity with bitter vetch roots showed low germination rates (average 2.90% ± standard error of the mean 0.13), ranging from 0.06% ± 0.04 to 5.66% ± 0.79 as a maximum with a highly significant genotypic effect (*p* < 0.0001). Accession Ve.34 showed the highest stimulating activity on broomrape germination (5.66% ± 0.79), followed by a set of 13 accessions including the susceptible control Ve.38, which ranged between 5.28% ± 0.42 and 4.04% ± 0.36. On the other hand, a set of 18 accessions (including Ve.5) stimulated low broomrape germination ranging from 2.09% ± 0.47 to 0.06% ± 0.04 (Table 1).

### 2.2. Field Screening

**Figure 1:**
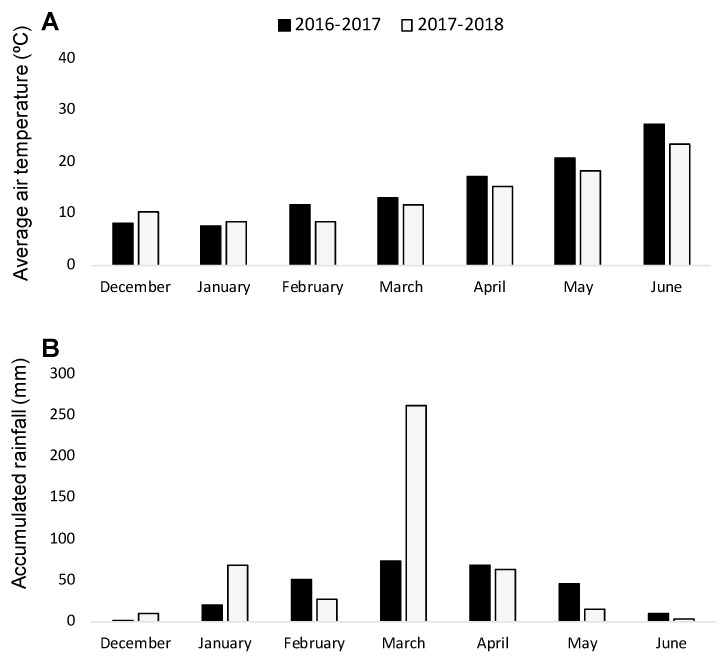
Average air temperature (**A**) and monthly rainfall (**B**) during the growing seasons 2016–2017 and 2017–2018.

With the aim of validating the results obtained in the rhizotron experiment, two consecutive field experiments were carried out during the seasons of 2016–2017 and 2017–2018. Weather conditions (Figure 1) were conducive for *O. crenata* infection and its emergence [7], as it was indicated by the *O. crenata* emergence on the susceptible faba bean checked (average of 2.85 ± 0.18 and of 7.05 ± 0.49 emerged *O. crenata* per faba bean plant during 2016–2017 and 2017–2018 seasons, respectively).

During the first growing season (2016–2017), a set of 17 accessions selected from the previous rhizotron experiment due to their contrasting resistance against *O. crenata* were evaluated under naturally infected soil conditions. Lines Ve.05, Ve.18, Ve.19, Ve.20, Ve.24, Ve.25, and Ve.26 were selected among the low inducers of *O. crenata* germination with lowest levels of tubercles formation in their roots. In addition, 10 lines including the susceptible accession Ve.38 with the highest stimulating activity of *O. crenata* germination and a higher number of tubercles were included as susceptible controls. As a resistant bitter vetch control, the cultivar Jabegote was selected due to its previously characterized incomplete resistance, but high productivity under *O. crenata* infestation [7] was used.

The average number of emerged *O. crenata* in all rhizotron-selected bitter vetch plants was 1.28 ± 0.13 ranging from 0.50 ± 0.27 in the most resistant accession to 3.26 ± 0.54 in the most susceptible accession. The bitter vetch accessions Ve.16 and Ve.38 showed significantly higher infection (2.28 ± 0.49 and 3.26 ± 0.54, respectively) than the control Jabegote (0.84 ± 0.46), while the rest of the rhizotron-selected bitter vetch accessions showed no significant differences in *O. crenata* emergence in comparison with Jabegote.

When the *O. crenata* infection of bitter vetch plants was standardized to the emerged *O. crenata* on the faba bean check rows surrounding each bitter vetch accession, *O. crenata* emergence on plants of the resistant control accession Jabegote was 16.33% ± 0.33, and on plants of the susceptible accession Ve.38, it was 129.33% ± 21.07. The average of *O. crenata* emergence in all rhizotron-selected bitter vetch accessions was 59.51% ± 6.12, ranging from 15.00% ± 5.00 in the accession Ve.24 to 153.00% ± 17.32 in the accession Ve.18. Among the rhizotron-selected bitter vetch accessions, all accessions showed significantly lower broomrape in comparison with the susceptible accession Ve.38 except for the accessions Ve.16, Ve.18, Ve.20, and Ve.30 (Table 2).

During the second growing season (2017–2018), a total of 6 accessions (Ve.5, Ve.19, Ve.24, Ve.25, Ve.26, and Ve.43), with low values of infection in 2016–2017, were selected and studied. In addition, the susceptible accession Ve.38 (as a negative control) and the resistant control cultivar Jabegote (as a positive control) were again studied under naturally infested-soil field conditions. The average number of emerged *O. crenata* per bitter vetch plant in all selected accessions was 0.98 ± 0.08 ranging from 0.56 ± 0.07 in the most resistant accessions to 1.72 ± 0.16 in the most resistant accessions (Table 3). The average of *O. crenata* emergence in bitter vetch referred to the faba bean check was 12.29% ± 1.25, ranging from 6.13% ± 0.01 in the most resistant accessions to 20.91% ± 2.60 in the most susceptible accessions. Besides the control Jabegote, the accessions Ve.24, Ve.19, and Ve.5 showed a significant low number of emerged *O. crenata* shoots, with Ve.24 being the accession with the lowest value. By contrast, the most susceptible accession in 2017–2018 field season was Ve.43 followed by Ve.25, Ve.38, and Ve.26 bitter vetch lines (Table 3).

In addition to *O. crenata* germination and development, several parameters describing the effects of *O. crenata* infection on bitter vetch productivity were assessed during the 2017–2018 field season as further indicators of the disease severity. The cumulative dry matter of all *O. crenata* plants emerged per bitter vetch plant averaged 1.72 g ± 0.22 and the average weight of individual *O. crenata* plants averaged 0.19 g ± 0.04. The relative parasite weight (ratio between parasite biomass and combined bitter-vetch–parasite biomass) averaged 0.29 ± 0.02. The aboveground cumulative *O. crenata* dry matter, the weight of individual parasites emerged as well as the relative emerged parasitic weight were not significantly different among lines. Symptoms of *O. crenata* damage were severe at flowering time in accessions Ve.25, Ve.38, and Ve.43, being dead or presenting flowering abortion (data not shown), which possibly indicate that a larger number of *O. crenata* plants attached to these bitter vetch accessions killed the host before emergence. The strong competition for resources by underground *O. crenata* plants before emergence in highly sensible accessions exhaust the resources killing both the host and the parasite before the end of their life cycles. The total emerged parasitic biomass per bitter vetch plant averaged from 1.28 ± 0.47 g/bitter vetch plant in accession Ve.38 (susceptible control) to 2.98 ± 1.31 g/bitter vetch plant in accession Ve.25. The weight of a single parasite growing per plant in each bitter vetch accession averaged from 0.09 ± 0.01 g/broomrape plant in accession Ve.38 to 0.42 ± 0.32 g/broomrape plant in accession Ve.24. The relative parasitic weight (ratio between parasite biomass and combined bitter-vetch–parasite biomass) averaged from 0.22 ± 0.01 in accession Ve.19 to 0.43 ± 0.07 in the susceptible accession Ve.38.

Bitter vetch productivity was also characterized as total host dry matter (vegetative and reproductive host aboveground tissues), which averaged 3.99 ± 0.48 g per plant, and host reproductive index (ratio between host reproductive dry matter and total aboveground host dry matter), which averaged 0.27 ± 0.04. Total host dry matter averaged from 1.67 ± 0.60 in line Ve.38 (susceptible) and 7.75 ± 0.93 in line Ve.19 (Table 3). Host reproductive index averaged from 0.00 in line Ve.38 (failure of seed setting) to 0.48 ± 0.05 in accession Ve.5 (Table 3).

### 2.3. In Vitro Germination Bioassay in Different Broomrape Species

**Figure 2:**
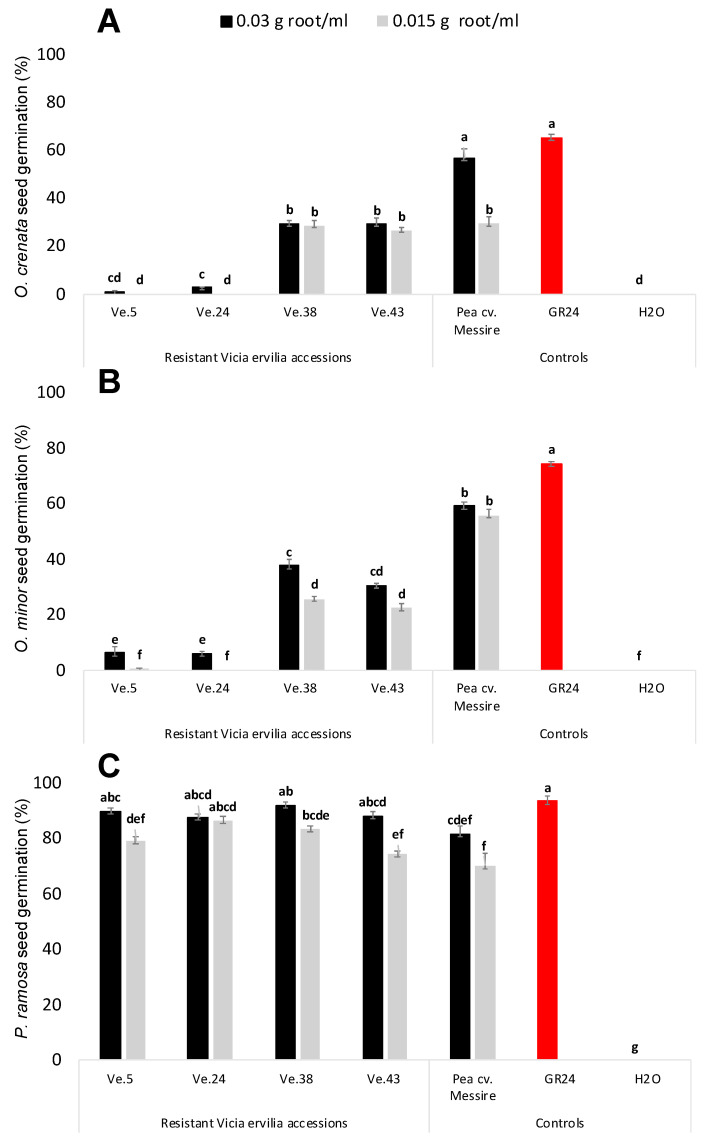
Effect of *Vicia ervilia* accession and concentration of hydroponically collected root exudates on stimulation of seed germination of (**A**) *O. crenata*, (**B**) *O. minor*, and (**C**) *P. ramosa*. Analysis of variance was applied to transformed replicate data. For each treatment, bars with different letters are significantly different according to the Tukey test (*p* < 0.05). Error bars represent the mean + s.e.

The stimulatory activity of root exudates of four rhizotron- and field-selected bitter vetch resistant accessions, e.g., Ve.5, Ve.24, Ve.38, and Ve.43, was studied in seeds of three broomrape species *O. crenata*, *O. minor*, and *P. ramosa* (Figure 2). In all cases, null germination was observed when seeds of the broomrape species were treated with distilled water (negative control). Significant effect in broomrape germination was observed for plant accession, (ANOVA, *p* < 0.0001) and broomrape species, (ANOVA, *p* < 0.0001), but not for root-exudate concentration. Root exudates of the highly resistant bitter vetch accessions Ve.5 and Ve.24 induced the lowest levels of *O. crenata* (1.01% ± 0.34 and 2.71% ± 0.22, respectively) and *O. minor* germination (6.3% ± 2.33 and 6.02% ± 0.84, respectively). Germination inducing activity of Ve.5 and Ve.24 was not statistically different than the germination induced by the negative control. The synthetic strigolactone GR24 followed by the susceptible cultivar of *P. sativum* cv. Messire, used as positive controls, induced the highest stimulatory activity on germination of *O. crenata* (56.77% ± 3.78 and 65.26% ± 1.30, respectively) and *O. minor* (59.12% ± 1.35 and 74.39% ± 0.98, respectively). Accessions Ve.38, Ve.43 and Ve.46 induced moderate levels of *O. crenata* and *O. minor* germination (Figure 2A,B). In addition to studying the stimulatory effect of bitter vetch root exudates on the legume-specialized broomrape species *O. crenata* and *O. minor*, we studied their effect in the broomrape species *P. ramosa*, which is a generalist parasite of many vegetable crops [8]. No significant effect was observed among treatments in germination induction. All accessions and controls studied in this work induced high stimulatory effects on seeds of *P. ramosa* (Figure 2C).

**Figure 3:**
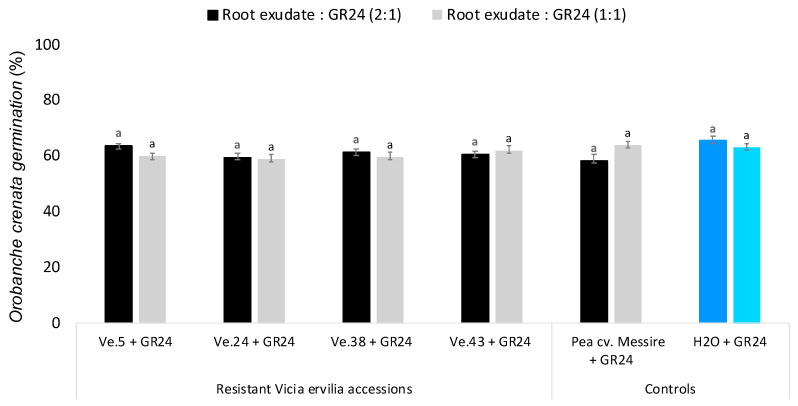
Effect of *Vicia ervilia* accession and concentration of hydroponically collected root exudates on inhibition of seed germination of *O. crenata*. Analysis of variance was applied to transformed replicate data. For each treatment, bars with different letters are significantly different according to the Tukey test (*p* < 0.05). Error bars represent the mean + s.e.

To discern whether the low germination activity of bitter vetch accessions Ve.5 and Ve.24 on seeds of *O. crenata* was due to low exudation of germination stimulants or exudation of germination inhibitors, root exudates of each plant accession were applied to *O. crenata* seeds mixed with the synthetic germination stimulant GR24 (Figure 3). None of the root exudates analyzed were actively inhibiting the germination-inducing activity of GR24 at the concentrations tested, revealing that the low germination activity observed in the bitter vetch accessions Ve.5 and Ve.24 was indeed due to low exudation of germination stimulants and not due to the exudation of germination inhibitors.

## 3. Discussion

Low germination-inducer genotypes of bitter vetch were successfully identified from our combination of rhizotron, field, and in vitro screenings of a germplasm collection, showing that resistance against the root parasitic plants *O. crenata* and *O. minor* based in low exudation of germination stimulants exists within existing phytogenetic resources. Therefore, selection of this trait is possible in breeding programs for broomrape resistance also in bitter vetch. Low induction of *O. crenata* germination in the identified resistant phenotypes is in accordance with low levels of root infection in rhizotron and field assays, as well as with seeds production.

Low induction of germination was effective against the legume-specialized broomrape species *O. minor* and *O. crenata*, but not against the generalist broomrape species *P. ramosa.* More than 25 host-derived germination stimulants have been identified in crops [32], and a parasite-recognition mechanism of host range has been found to be achieved by means of a germination tuning between parasitic and host species through: (i) exudation of host species-specific cocktails of germination stimulants and (ii) broomrape species-specific variation in germination responses to different host root exudates [13,24,33,34,35]. Root exudates of bitter vetch accessions Ve.5 and Ve.24 were low in the specific stimulants that induce germination responses of the legume-specific broomrape species *O. crenata* and *O. minor*, but not in the stimulants that induce germination in *P. ramosa*. Therefore, these accessions could have additional applications as trap crops inducing suicidal germination of incompatible broomrape species, as was previously found in pea [36]. An alternative explanation of the low germination activity in root exudates of the accessions Ve.5 and Ve.24 could be their exudation of germination inhibitors specifically active on *O. crenata* and *O. minor*. However, mixtures of exudates of the low-inducer bitter vetch accessions with the germination stimulant GR24 were active in inducing the germination of *O. crenata*, and therefore, we ruled out the hypothesis of exudation of germination inhibitors. Absence of inhibitory activity was also found in low inducer-genotypes of faba bean and later confirmed that the low induction effect was due to low exudation of strigolactones [23,24].

The chemical class of germination stimulants exuded by bitter vetch roots and the inheritance of this trait are unknown and should be studied in future research as have been studied for other crop species showing this resistance mechanism [24,37,38]. Broomrape resistance achieved by this mechanism was complete and could be pyramided with other resistance mechanisms acting at subsequent steps in the infection process of *O. crenata* in bitter vetch identified in previous work [7]. This could lead to increased resistance with prolonged durability against *O. crenata* infection [12].

An important feature in bitter vetch sources of parasitic weed resistance is the ability to be productive in soils highly infested by *O. crenata* infection [7]. In some legume species, accessions with levels of resistance are not able to yield to their potential when they are infected by a reduced number of *O. crenata* plants due to the strong competition for nutrients created by the parasite even at low levels of infection [10]. Measurements of biomass made in this work indicated that bitter vetch accessions with resistance based in reduced levels of *O. crenata* germination produced more biomass in comparison with the susceptible accessions Ve.43 and Ve.38, both in terms of total host dry matter and host reproductive index. This increase in biomass was visually obvious but not statistically significant, except for the bitter vetch accession Ve.19 showing the highest biomass. An *O. crenata* infection reduces the host biomass mainly at the expense of the host reproductive tissue [10]. The capacity to set seeds in the resistant accessions was significantly higher in the resistant accessions than in the susceptible accessions Ve.43 and Ve.38, showing the resistant accession Ve.5 the highest reproductive index under parasitic infection. The experimental design used in this work is a method specifically designed for the investigation of levels of *O. crenata* infection [7,39,40], showing solid results in the field identification of resistant genotypes. However, the results are less strong for biomass measurements as they are performed in rows. Further experiments specifically designed for yield measurements are needed to validate the increase in yield observed in our work. The bitter vetch accessions described in this work represent good bitter vetch candidate resources for breeding programs required to promote the use of this legume.

## 4. Materials and Methods

Accessions belonging to a bitter vetch (*V. ervilia*) germplasm collection was studied in order to identify lines with low ability to induce *O. crenata* germination and subsequently low infection levels. This material was provided by Plant Genetic Resources Center (CRF, Madrid, Spain), the Genetic Resources Unit of International Center for Agricultural Research in the Dry Areas, (ICARDA, Aleppo, Syria), and Legumes Genebank (BGLI, at IFAPA, Cordoba, Spain). For rhizotron experiments, broomrape seeds were collected from dry inflorescences of *O. crenata* plants growing on *Vicia faba* plants using a 0.6 mm mesh-size sieve (Filtra, Barcelona, Spain) and stored dry in the dark at 4 °C for three months before their use.

### 4.1. Rhizotron Screening

Host–parasite interactions were studied in 39 bitter vetch lines (Table 1) through a rhizotron experiment, following the methodology described by Pérez-de-Luque et al. [39]. Bitter vetch and *O. crenata* seeds were previously surface disinfected with sodium hypochlorite solution 5% *w*/*v*. Bitter vetch seedlings and approximately 6 mg of *O. crenata* seeds were simultaneously sown on a sheet of glass-fiber filter paper (GFFP, Whatman G/A, Whatman International Ltd., Maidstone, UK) inside 12 x12 cm^2^ squared Petri dishes containing perlite. The roots of bitter vetch and *O. crenata* seeds were developed on the GFFP sheets, and the aerial part of the host plant was developed outside of the dish through a hole in the dish. Petri dishes were wrapped in aluminum foil to maintain *O. crenata* seeds in the dark so that the preconditioning and infection took place adequately. The system was watered and vertically stored in a growth chamber (21 °C; 16 h light). Plants received Hoagland’s nutrient solution [41]. Ten independent plates (each a randomized block) were set for each accession. Percentages of seed germination were determined based on 400 *O. crenata* seeds per plate (100 seeds in each section of the four analysis fields established per plate). The host root length was determined by the intercept method of Tennant [42].

### 4.2. Field Screening

In order to validate the results obtained in the rhizotron experiment, a set of contrasting lines were assessed in a field naturally infested with *O. crenata* seeds at the IFAPA Alameda del Obispo Center (Cordoba, Spain) during the seasons of 2016–2017 and 2017–2018.

The first field experiment was performed during the season 2016–2017 with 17 bitter vetch accessions, which behaved in a contrasting way regarding their ability to induce *O. crenata* gemination and tubercle development in the rhizotron assay. The sowing date was the December 23, 2016. Each bitter vetch accession was sown in a row of 0.5 m with 25 seeds per row and a distance between rows of 1 m with three replicates in a randomized complete block design. Since there was no information about bitter vetch-susceptible lines, each bitter vetch row was alternately surrounded by rows of the susceptible control faba bean cv Prothabon [43]. Broomrape infection was assessed at crop maturity counting the number of emerged broomrapes per plant within each row. In order to avoid the error caused by the heterogeneous distribution of broomrape seeds across the trial, the number of emerged broomrapes per host plant within each row was standardized as “broomrape emergence referred to the check” as a percentage of the mean value of the four surrounding rows of faba bean control [40]. Bitter vetch cv Jabegote was also included in the study due to both its moderate tolerance to *O. crenata* and its previously characterized yield data [7].

The second field experiment was conducted during the growing season 2017–2018 in order to obtain more accurate and detailed information of the results previously observed in the first field trial. With this end, the seven accessions showing the lowest values of “broomrape emergence referred to the check” across the first field experiment were sown the December 22, 2017. As in the previous experiment, each line was sown in a row of 0.5 m with 25 seeds per row in three repetitions. The susceptible control faba bean cv Prothabon was included as a control as described above in order to normalize the broomrape infection data for each bitter vetch test row, as well as bitter vetch cv Jabegote. As before, the number of emerged broomrapes per bitter vetch row was standardized as “broomrape emergence referred to the check” as a percentage of the mean value of the four surrounding rows of faba bean control [40].

In addition to the final number of emerged broomrapes, the level of severity caused by the parasite was studied in the most contrasting lines by using additional parameters as described by Fernández-Aparicio et al. [10]: (i) cumulative parasitic dry matter per host plant (total dry biomass of emerged broomrapes per host plant); (ii) average weight of individual parasites in each host plant (cumulative broomrape dry matter/number of broomrapes per host plant); (iii) total host dry matter (vegetative and reproductive bitter vetch aboveground tissues); (iv) combined biomass (host and parasite biomass); (v) relative parasitic weight (ratio between parasite biomass and combined biomass); and (vi) host reproductive index (ratio between host reproductive dry matter and total aboveground host dry matter).

### 4.3. In Vitro Germination Bioassays

#### 4.3.1. Identification of Germination-Stimulatory Activity

The root activity of resistant bitter vetch accessions Ve.5, Ve.24, Ve.38, and Ve.43 on broomrape seed germination was compared with the root activity of two positive controls: pea (*Pisum sativum*) cv. Messire and the synthetic strigolactone GR24 (10^−6^ M), and one negative control (distilled water), according to previous protocols [24,33]. Due to legume root exudates having been shown to induce species-specific stimulation of *Orobanche* seed germination [33,44], seeds of three broomrape species (*O. crenata*, population collected in faba bean in Spain, *O. minor* population collected in red clover in France and *P. ramosa* population collected in tobacco in France) were used to identify differences in germination-induction activity for each plant accession.

Seeds of bitter vetch and pea were surface sterilized with 4% sodium hypochlorite containing 0.02% Tween 20, rinsed three times with sterile distilled water and placed on moistened filter paper inside Petri dishes to allow for germination. Four days later, the germinated seeds were transferred to pots filled with sterile perlite and cultivated during 31 days in a growth chamber (23/20 °C, 16/8 h day/night). Plants received Hoagland’s nutrient solution [41] modified at one-quarter strength once per week. Legume plants were removed from the perlite, their roots carefully washed and individually placed in 50 mL tubes by immersing the roots for 48 h in sterile distilled water, allowing them to release the root exudates. The root exudate solutions of each legume plant were collected individually, and the total legume root contained in each tube weighed. In order to make valid comparisons across legume accessions and plants, the root exudate solution was adjusted with sterile distilled water to achieve equivalent concentrations of 0.03 and 0.015 g of legume root fresh weight/mL of root-exudate solution.

Broomrape seeds were surface sterilized by immersion in 0.5% (*w*/*v*) NaOCl and 0.02% (*v*/*v*) Tween 20 for 5 min, rinsed thoroughly with sterile distilled water, and dried in a laminar airflow cabinet. Approximately 100 seeds of each broomrape species were placed separately in 132 glass-fiber filter paper (GFFP) 9 mm diameter-disks moistened with 100 μL of sterile distilled water and placed inside Petri dishes in incubators at 23 °C for 10 days to allow for seed conditioning. GFFP disks containing conditioned broomrape were transferred on a sterile sheet of filter paper to remove the excess of water and transferred to new 10 cm sterile Petri dishes. Differences in germination induction were studied by applying triplicate aliquots of 50 μL of root exudate collected from each legume plant (three plants per legume accession). Seeds were stored in the dark at 23 °C for 7 d to allow for germination. The germination was determined using a stereoscopic microscope by counting the number of germinated seeds on 100 seeds for each GFFP disk.

#### 4.3.2. Identification of Germination-Inhibitory Activity

A germination-inhibition-activity assay was performed according to previous protocol [23,45] in order to rule out the possibility that the lack of germination-induction activity in bitter vetch accessions was due to exudation of germination inhibitors instead of the lack of exudation of germination stimulants. The root exudates of bitter vetch accessions Ve.5, Ve.24, Ve.38, and Ve.43, and the control pea cv Messire were tested on *O. crenata* seeds mixed with the synthetic germination stimulant GR24 and compared with the germination induced by an equivalent concentration of GR24 without root exudate. The *O. crenata* seeds were surface sterilized, spread in GFFP disks, and conditioned for 10 days as described above. Fifty microliter aliquots of each root-exudate GR24 mixture were applied to each GFFP disc containing the conditioned seeds. Petri dishes were sealed with Parafilm and stored in the dark at 22 °C for 7 days to promote germination and radicle growth. The percentage of seed germination were established for each GFFP disc, as described above, in order to score levels of root-exudate-mediated inhibition.

### 4.4. Statistical Analysis

The experimental design was randomized in complete blocks. Percentage data were transformed with arcsin (√(x/100) before analysis. Analysis of variance (one-way ANOVA) was applied to replicate data with accession as the main factor using Statistix 9.1 software (Analytical software, Tallahassee, FL, USA). The significance of mean differences among accessions was evaluated by Tukey test. Null hypothesis was rejected at the level of 0.05.

## 5. Conclusions

Resistant phenotypes inducing low induction of *O. crenata* germination are present in bitter vetch (*V. ervilia*) germplasm as revealed by experiments based on rhizotron, field, and in vitro screenings. In the present work, two novel accessions with limited capacity for stimulating *O. crenata* seeds germination and exhibiting a high level of field resistance, as well as high productivity have been described. These results show a novel resistant source in bitter vetch to give the possibility of contributing to *O. crenata* management through breeding programs and to promote the use of this legume as a source of protein for animal feed in low-input cropping systems. New experiments with these lines in order to elucidate the genes involved in the resistance mechanism would be of maximum interest and will be performed in the future.

## Figures and Tables

**Table 1:** Broomrape seed germination, number of broomrape tubercles per plant and host root length in bitter vetch accessions. ± (Standard error of the mean). Analysis of variance was applied to replicate data. Accessions with different letters are significantly different according to the Tukey test (*p* = 0.05).

Accession	Code ICARDA	Origin	Broomrape Seed Germination (%)	N.º of Broomrape Tubercles per Plant	Host Root Length (cm)
BGLI-Ve.005	IFVE 2842	Unknown	0.32 ± 0.20 ^ab^	0.57 ± 0.30 ^ab^	535 ± 36.86 ^fghijk^
BGLI-Ve.006	IFVE 2847	Unknown	1.68 ± 0.41 ^bcdefg^	2.29 ± 0.68 ^abcd^	468 ± 46.56 ^bcdefghij^
BGLI-Ve.009	IFVE 2943	Unknown	1.25 ± 0.29 ^abcdef^	2.50 ± 0.50 ^abcde^	267 ± 47.07 ^abc^
BGLI-Ve.010	IFVE 3030	Unknown	2.00 ± 0.43 ^bcdefghi^	2.57 ± 1.17 ^abcde^	523 ± 81.69 ^defghijk^
BGLI-Ve.011	IFVE 2849	Unknown	1.64 ± 0.76 ^abcde^	3.29 ± 1.17 ^abcde^	437 ± 25.11 ^abcdefghij^
BGLI-Ve.012	IFVE 2542	Unknown	4.75 ± 1.28 ^efghi^	4.17 ± 1.85 ^abcdef^	412 ± 56.94 ^abcdefghij^
BGLI-Ve.013	IFVE4654	Unknown	3.34 ± 0.46 ^defghi^	1.88 ± 0.48 ^abcd^	585 ± 52.26 ^jk^
BGLI-Ve.014	IFVE 4657	Unknown	4.43 ± 0.78 ^efghi^	1.86 ± 0.94 ^abc^	408 ± 33.17 ^abcdefghij^
BGLI-Ve.015	IFVE 4658	Unknown	1.35 ± 0.69 ^abcde^	1.20 ± 0.97 ^abc^	551 ± 46.82 ^hijk^
BGLI-Ve.016		Unknown	2.88 ± 0.71 ^cdefghi^	5.75 ± 1.41 ^abcdefg^	442 ± 39.35 ^abcdefghij^
BGLI-Ve.017	SEL 2510	Chipre	1.32 ± 0.44 ^abcde^	0.86 ± 0.55 ^abc^	720 ± 63.02 ^k^
BGLI-Ve.018	SEL 2511	Chipre	0.06 ± 0.0 ^a^	0.25 ± 0.16 ^a^	570 ± 47.98 ^ijk^
BGLI-Ve.019	SEL 2512	Chipre	0.71 ± 0.34 ^abc^	1.00 ± 0.85 ^abc^	340 ± 28.95 ^abcdefghi^
BGLI-Ve.020	SEL 2513	Chipre	1.94 ± 0.36 ^bcdefghi^	3.50 ± 0.80 ^abcde^	541 ± 28.57 ^ghijk^
BGLI-Ve.021	SEL 2515	Chipre	1.19 ± 0.53 ^abcd^	1.75 ± 0.75 ^abc^	346 ± 69.34 ^abcdefghij^
BGLI-Ve.022	SEL 2516	Chipre	2.56 ± 0.62 ^cdefghi^	2.63 ± 0.71 ^abcde^	393 ± 40.40 ^abcdefghij^
BGLI-Ve.023	SEL 2517	Chipre	0.78 ± 0.15 ^abcd^	1.63 ± 0.46 ^abc^	436 ± 29.27 ^abcdefghij^
BGLI-Ve.024	SEL 2518	Siria	1.56 ± 0.59 ^abcde^	1.25 ±0.45 ^abc^	527 ± 40.35 ^efghijk^
BGLI-Ve.025	SEL 2519	Siria	1.25 ± 0.72 ^abcd^	1.88 ± 0.90 ^abcd^	216 ± 16.95 ^a^
BGLI-Ve.026	SEL 2520	Siria	1.65 ± 0.34 ^bcdefgh^	2.75 ± 1.06 ^abcde^	284 ± 18.07 ^abcd^
BGLI-Ve.027	SEL 2522	Chipre	2.00 ± 0.24 ^bcdefghi^	4.43 ± 0.65 ^abcdefg^	504 ± 67.73 ^cdefghijk^
BGLI-Ve.028	SEL 2563	Siria	3.09 ± 0.45 ^defghi^	3.00 ± 0.63 ^abcde^	240 ± 18.94 ^ab^
BGLI-Ve.029	SEL 2644	Bulgaria	2.06 ± 0.43 ^bcdefghi^	2.63 ± 1.21 ^abcde^	301 ± 20.91 ^abcdef^
BGLI-Ve.030	SEL 2647	Bulgaria	2.72 ± 1.10 ^bcdefghi^	3.13 ± 1.64 ^abcde^	390 ± 34.94 ^abcdefghij^
BGLI-Ve.031	SEL 2648	Bulgaria	2.44 ± 0.28 ^cdefghi^	2.00 ± 0.93 ^abcd^	402 ± 50.40 ^abcdefghij^
BGLI-Ve.032		Bulgaria	4.63 ± 0.56 ^fghi^	6.00 ± 1.15 ^abcdefg^	236 ± 14.63 ^ab^
BGLI-Ve.033		Unknown	4.04 ± 0.36 ^efghi^	4.86 ± 0.51 ^abcdefg^	233 ± 15.21 ^ab^
BGLI-Ve.034		Spain	5.66 ± 0.79 ^i^	10.63 ± 2.46 ^g^	248 ± 19.98 ^ab^
BGLI-Ve.035		Spain	5.06 ± 0.59 ^ghi^	6.63 ± 1.03 ^bcdefg^	559 ± 83.52 ^ijk^
BGLI-Ve.036		Spain	4.50 ± 1.36 ^efghi^	6.40 ± 1.57 ^abcdefg^	293 ± 25.79 ^abcde^
BGLI-Ve.037		Spain	5.25 ± 0.47 ^ghi^	8.71 ± 1.46 ^efg^	257 ± 5.78 ^ab^
BGLI-Ve.038		Unknown	4.66 ± 0.54 ^fghi^	8.13 ± 1.09 ^defg^	316 ± 41.95 ^abcdefgh^
BGLI-Ve.039		Spain	5.28 ± 0.42 ^hi^	10.25 ± 1.81 ^fg^	232 ± 46.06 ^ab^
BGLI-Ve.040		Unknown	5.10 ± 0.62 ^ghi^	6.40 ± 0.98 ^abcdefg^	367 ± 60.25 ^abcdefghij^
BGLI-Ve.041		Spain	2.09 ± 0.47 ^bcdefghi^	1.75 ± 0.70 ^abc^	342 ± 27.03 ^abcdefghi^
BGLI-Ve.042		Unknown	5.13 ± 0.71 ^ghi^	5.75 ± 1.26 ^abcdefg^	306 ± 31.02 ^abcdefg^
BGLI-Ve.043		Unknown	3.28 ± 0.69 ^cdefghi^	6.25 ± 1.73 ^abcdefg^	291 ± 53.58 ^abcde^
BGLI-Ve.044		Unknown	4.94 ± 0.87 ^ghi^	6.88 ± 0.69 ^cdefg^	224 ± 23.09 ^a^
BGLI-Ve.045		Spain	4.84 ± 0.76 ^ghi^	6.25 ± 1.29 ^abcdefg^	238 ± 14.98 ^ab^

There was also a positive correlation (r = 0.7425, *p* < 0.0001) between the germination percentage and the number of tubercles per plant. Thus, bitter vetch accessions more efficient stimulating broomrape germination also showed higher number of tubercles in their roots. In accordance with this, the bitter vetch accession Ve.34 with higher germination inducing activity also presented the higher number of *O. crenata* tubercles per plant (10.63 ± 2.46), followed by the accessions Ve.39 and Ve.37 and the susceptible control Ve.38 with 10.25 ± 1.81, 8.71 ± 1.46, and 8.13 ± 1.09 tubercles per plant, respectively. In contrast, the low *O. crenata* germination-inducing genotypes were included in a set of 23 accessions that showed the lowest levels of tubercles formation in their roots (average of 2.064 ± 0.193) (Table 1).

**Table 2:** Number of broomrape as well as emerged *O. crenata* in field as percent of faba bean control, on selected bitter vetch lines and the commercial bitter vetch variety Jabegote during the season of 2016–2017. ± (standard error of the mean). Analysis of variance was applied to replicate data. Accessions with different letters are significantly different according to the Tukey test (*p* = 0.05).

Accession	Number of *O. crenata* per Bitter Vetch Plant	*O. crenata* Emergence in Bitter Vetch Referred to the Faba Bean Check (%)
BGLI-Ve.5	1.17 ± 0.56 ^ab^	37.33 ± 2.03 ^ab^
BGLI-Ve.12	1.48 ± 0.62 ^ab^	32.33 ± 8.33 ^a^
BGLI-Ve.16	2.28 ± 0.49 ^ab^	76.33 ± 8.95 ^abcd^
BGLI-Ve.18	1.25 ± 0.14 ^ab^	153.00 ± 17.32 ^e^
BGLI-Ve.19	0.50 ± 0.27 ^a^	19.33 ± 1.45 ^a^
BGLI-Ve.20	1.74 ± 0.46 ^ab^	101.33 ± 3.18 ^bcde^
BGLI-Ve.24	0.56 ± 0.18 ^a^	15.00 ± 5.00 ^a^
BGLI-Ve.25	0.98 ± 0.51 ^ab^	20.33 ± 4.33 ^a^
BGLI-Ve.26	0.73 ± 0.15 ^a^	28.67 ± 2.73 ^a^
BGLI-Ve.30	1.20 ± 0.67 ^ab^	125.33 ± 20.50 ^cde^
BGLI-Ve.34	0.84 ± 0.37 ^a^	48.67 ± 19.33 ^ab^
BGLI-Ve.35	1.64 ± 0.34 ^ab^	61.67 ± 3.76 ^abc^
BGLI-Ve.36	0.73 ± 0.45 ^a^	48.33 ± 11.84 ^ab^
BGLI-Ve.38	3.26 ± 0.54 ^b^	129.33 ± 21.07 ^de^
BGLI-Ve.39	1.27 ± 0.70 ^ab^	47.67 ± 19.23 ^ab^
BGLI-Ve.43	0.83 ± 0.27 ^a^	37.33 ± 3.18 ^ab^
BGLI-Ve.44	1.72 ± 0.34 ^ab^	58.00 ± 14.73 ^ab^
Jabegote	0.84 ± 0.46 ^a^	16.33 ± 0.33 ^a^

**Table 3:** Number of emerged broomrapes per host plant, broomrape emergence in bitter vetch referred to the faba bean check, as well as parameters of broomrape infection on selected bitter vetch lines and the commercial bitter vetch variety Jabegote during the season of 2017–2018. ± (standard error of the mean). Analysis of variance was applied to replicate data. Accessions with different letters are significantly different according to the Tukey test (*p* = 0.05).

Accession	Number of *O. crenata* per Plant	*O. crenata* Emergence Referred to the Faba Bean Check (%)	Total *O. crenata* Emerged Dry Matter per Bitter Vetch Plant (g)	Average Individual *O. crenata* Dry Matter (g)	Relative Emerged Parasitic Weight	Total Host Dry Matter (g)	Host Reproductive Index
BGLI-Ve.5	1.10 ± 0.01 ^c^	11.87 ± 0.06 ^ab^	1.56 ± 0.78 ^a^	0.10 ± 0.06 ^a^	0.23 ± 0.12 ^a^	4.43 ± 0.54 ^ab^	0.48 ± 0.05 ^d^
BGLI-Ve.19	0.56 ± 0.07 ^a^	7.90 ± 0.01 ^ab^	1.52 ± 0.43 ^a^	0.13 ± 0.01 ^a^	0.22 ± 0.01 ^a^	7.75 ± 0.93 ^b^	0.35 ± 0.01 ^cd^
BGLI-Ve.24	0.59 ± 0.15 ^ab^	6.13 ± 0.01 ^a^	1.57 ± 0.80 ^a^	0.42 ± 0.32 ^a^	0.22 ± 0.04 ^a^	2.08 ± 1.29 ^a^	0.23 ± 0.01 ^abc^
BGLI-Ve.25	0.84 ± 0.02 ^abc^	15.44 ± 0.03 ^bc^	2.98 ± 1.31 ^a^	0.30 ± 0.18 ^a^	0.24 ± 0.02 ^a^	3.40 ± 1.98 ^ab^	0.41 ± 0.09 ^cd^
BGLI-Ve.26	0.88 ± 0.10 ^abc^	12.88 ± 0.03 ^abc^	2.08 ± 0.56 ^a^	0.19 ± 0.07 ^a^	0.31 ± 0.05 ^a^	4.45 ± 0.39 ^ab^	0.27 ± 0.01 ^bcd^
BGLI-Ve.38	1.08 ± 0.08 ^bc^	15.05 ± 0.01 ^abc^	1.28 ± 0.47 ^a^	0.09 ± 0.01^a^	0.43 ± 0.07 ^a^	1.67 ± 0.60 ^a^	0.00 ± 0.00 ^a^
BGLI-Ve.43	1.72 ± 0.16 ^d^	20.91 ± 0.03 ^c^	1.64 ± 0.70 ^a^	0.18 ± 0.06 ^a^	0.36 ± 0.02 ^a^	3.59 ± 0.68 ^ab^	0.04 ± 0.04 ^ab^
Jabegote	1.11 ± 0.11 ^c^	8.17 ± 0.00 ^ab^	1.54 ± 0.54 ^a^	0.15 ± 0.03 ^a^	0.34 ± 0.04 ^a^	4.34 ± 1.48 ^ab^	0.37 ± 0.08 ^cd^

## References

[1] Zohary D., Hopf M. (2000). Domestication of Plants in the Old World.

[2] Ladizinsky G. (1998). Plant Evolution under Domestication.

[3] Kaplan M., Kokten K., Uzun S. (2014). Fatty acid and metal composition of the seeds of Vicia ervilia varieties from Turkey. Chem. Nat. Compd..

[4] Ministry of Agriculture and Fisheries and Food (2019). Agri-Food Statistics Yearbook. https://www.mapa.gob.es/es/estadistica/temas/publicaciones/anuario-de-estadistica/2019/default.aspx?parte=3&capitulo=07&grupo=2&seccion=8.

[5] Enneking D., Lahlou A., Noutfia A., Bounejmate M. (1995). A note on *Vicia ervilia* cultivation utilisation and toxicity in Morocco. Al Awamia.

[6] Karadavut U., Bakoglu A., Tutar H., Kokten K., Yilmaz H.S. (2017). Prediction of dry matter accumulation in bitter vetch. Legume Res..

[7] González-Verdejo C.I., Fernández-Aparicio M., Córdoba E.M., Nadal S. (2020). Identification of *Vicia ervilia* germplasm resistant to *Orobanche crenata*. Plants.

[8] Parker C., Joel D.M., Gressel J., Musselman L.J. (2013). The parasitic weeds of the Orobanchaceae. Parasitic Orobanchaceae.

[9] Rubiales D., Fernández-Aparicio M. (2012). Innovations in parasitic weeds management in legume crops. Agron. Sustain. Dev..

[10] Fernández-Aparicio M., Flores F., Rubiales D. (2016). The effect of *Orobanche crenata* infection severity in faba bean, field pea and grass pea productivity. Front. Plant Sci..

[11] Fernández-Aparicio M., Sillero J.C., Rubiales D. (2009). Resistance to broomrape in wild lentils (*Lens* spp.). Plant Breeding.

[12] Fernández-Aparicio M., Delavault P., Timko M. (2020). Management of infection by parasitic weeds: A review. Plants.

[13] Xie X., Yoneyama K., Yoneyama K. (2010). The strigolactone story. Annu. Rev. Phytopathol..

[14] Joel D.M., Chaudhuri S.K., Plakhine D., Ziadna H., Steffens J.C. (2011). Dehydrocostus lactone is exuded from sunflower roots and stimulates germination of the root parasite *Orobanche cumana*. Phytochemistry.

[15] Chang M., Netzly D.G., Butler L.G., Lynn D.G. (1986). Chemical regulation of distance: Characterization of the first natural host germination stimulant for *Striga asiatica*. J. Am. Chem. Soc..

[16] Evidente A., Fernández-Aparicio M., Cimmino A., Rubiales D., Andolfi A., Motta A. (2009). Peagol and peagoldione, two new strigolactone-like metabolites isolated from pea root exudates. Tetrahedron Lett..

[17] Evidente A., Cimmino A., Fernández-Aparicio M., Rubiales D., Andolfi A., Melck D. (2011). Soyasapogenol B and *trans*-22-dehydrocampesterol from common vetch (*Vicia sativa* L.) root exudates stimulate broomrape seed germination. Pest Manag. Sci..

[18] Evidente A., Cimmino A., Fernández-Aparicio M., Andolfi A., Rubiales D., Motta A. (2010). Polyphenols, including the new peapolyphenols A− C, from pea root exudates stimulate *Orobanche foetida* seed germination. J. Agric. Food Chem..

[19] Auger B., Pouvreau J.B., Pouponneau K., Yoneyama K., Montiel G., Le Bizec B., Yoneyama K., Delavault P., Delourme R., Simier P. (2012). Germination stimulants of *Phelipanche ramosa* in the rhizosphere of *Brassica napus* are derived from the glucosinolate pathway. Mol. Plant- Microbe Interact..

[20] Yoder J.I., Scholes J.D. (2010). Host plant resistance to parasitic weeds; recent progress and bottlenecks. Curr. Opin. Plant Biol..

[21] Fernández-Aparicio M., Westwood J.H., Rubiales D. (2011). Agronomic, breeding and biotechnological approaches for parasitic plant management by manipulating strigolactone levels in agricultural soils. Botany.

[22] Abbes Z., Kharrat M., Simier P., Chaıbi W. (2007). Characterisation of resistance to crenate broomrape (*Orobanche crenata*) in a new small seeded line of Tunisian faba beans. Phytoprotection.

[23] Fernández-Aparicio M., Moral A., Kharrat M., Rubiales D. (2012). Resistance against broomrapes (*Orobanche* and *Phelipanche* spp.) in faba bean (*Vicia faba*) based in low induction of broomrape seed germination. Euphytica.

[24] Fernández-Aparicio M., Kisugi T., Xie X., Rubiales D., Yoneyama K. (2014). Low strigolactone root exudation: A novel mechanism of broomrape (*Orobanche* and *Phelipanche* spp.) resistance available for faba bean breeding. J. Agric. Food Chem..

[25] El-Halmouch Y., Benharrat H., Thalouarn P. (2006). Effect of root exudates from different tomato genotypes on broomrape (*O. aegyptiaca*) seed germination and tubercle development. Crop Prot..

[26] Dor E., Alperin B., Wininger S., Ben-Dor B., Somvanshi V.S., Koltai H., Kapulnik Y., Hershenhorn J. (2010). Characterization of a novel tomato mutant resistant to *Orobanche* and *Phelipanche* spp. weedy parasites. Euphytica.

[27] Pavan S., Schiavulli A., Marcotrigiano A.R., Bardaro N., Bracuto V., Ricciardi F., Charnikova T., Lotti C., Bouwmeester H., Ricciardi L. (2016). Characterization of low-strigolactone germplasm in pea (*Pisum sativum* L.) resistant to crenate broomrape (*Orobanche crenata* Forsk.). Mol. Plant Microbe Interact..

[28] Jamil M., Charnikhova T., Houshyani B., van Ast A., Bouwmeester H.J. (2012). Genetic variation in strigolactone production and tillering in rice and its effect on *Striga hermonthica* infection. Planta.

[29] Gobena D., Shimels M., Rich P.J., Ruyter-Spira C., Bouwmeester H., Kanuganti S., Mengiste T., Ejeta G. (2017). Mutation in sorghum low germination stimulant 1 alters strigolactones and causes *Striga* resistance. Proc. Natl. Acad. Sci. USA.

[30] Labrousse P., Arnaud M.C., Serieys H., Bervillé A., Thalouan P. (2001). Several mechanisms are involved in resistance of *Helianthus* to *Orobanche cumana* Wallr. Ann. Bot..

[31] Serghini K., Pérez-de-Luque A., Castejón-Muñoz M., García-Torres L., Jorrín J.V. (2001). Sunflower (*Helianthus annuus* L.) response to broomrape (*Orobanche cernua* Loefl.) parasitism: Induced synthesis and excretion of 7-hydroxilated simple coumarins. J. Exp. Bot..

[32] Aliche E.B., Screpanti C., De Mesmaeker A., Munnik T., Bouwmeester H.J. (2020). Science and application of strigolactones. New Phytol..

[33] Fernández-Aparicio M., Flores F., Rubiales D. (2009). Recognition of root exudates by seeds of broomrape (*Orobanche* and *Phelipanche*) species. Ann. Bot..

[34] Fernández-Aparicio M., Yoneyama K., Rubiales D. (2011). The role of strigolactones in host specificity of *Orobanche* and *Phelipanche* seed germination. Seed Sci. Res..

[35] Conn C.E., Bythell-Douglas R., Neumann D., Yoshida S., Whittington B., Westwood J.H., Shirasu K., Bond C.S., Dyer K.A., Nelson D.C. (2015). Convergent evolution of strigolactone perception enabled host detection in parasitic plants. Science.

[36] Fernández-Aparicio M., Rubiales D. (2012). Differential response of pea (*Pisum sativum*) to *Orobanche crenata*, *O. foetida* and *Phelipanche aegyptiaca*. Crop Prot..

[37] Ramaiah K.V., Chidley V.L., House L.R. (1990). Inheritance of *Striga* seed-germination stimulant in sorghum. Euphytica.

[38] Vogler R.K., Ejeta G., Butler L.G. (1996). Inheritance of low production of *Striga* germination stimulant in sorghum. Crop Sci..

[39] Pérez-de-Luque A., Jorrín J., Cubero J.I., Rubiales D. (2005). *Orobanche crenata* resistance and avoidance in pea (*Pisum* spp.) operate at different developmental stages of the parasite. Weed Res..

[40] Fernández-Aparicio M., Flores F., Rubiales D. (2012). Escape and true resistance to crenate broomrape (*Orobanche crenata* Forsk.) in grass pea (*Lathyrus sativus* L.) germplasm. Field Crop. Res..

[41] Hoagland D.R., Arnon D.I. (1950). The Water-culture Method for Growing Plants without Soil.

[42] Tennant D. (1975). A test of a modified line intersect method of estimating root length. J. Ecol..

[43] Rubiales D., Pérez-de-Luque A., Cubero J.I., Sillero J.C. (2003). Crenate broomrape (*Orobanche crenata*) infection in field pea cultivars. Crop Prot..

[44] Fernández-Aparicio M., Andolfi A., Evidente A., Pérez-de-Luque A., Rubiales D. (2008). Fenugreek root exudates show species-specific stimulation of *Orobanche* seed germination. Weed Res..

[45] Cimmino A., Fernández-Aparicio M., Andolfi A., Basso S., Rubiales D., Evidente A. (2014). Effect of fungal and plant metabolites on broomrapes (*Orobanche* and *Phelipanche* spp.) seed germination and radicle growth. J. Agric. Food Chem..

